# Optimization of braided stent design for cerebral aneurysms: the role of wire cross-sectional geometry

**DOI:** 10.3389/fbioe.2025.1643750

**Published:** 2025-08-28

**Authors:** Aohua Zhang, Xinru Li, Zhengbiao Yang, Yutang Xie, Tao Wu, Yanru Xue, Yanqin Wang, Yongwang Zhao, Weiyi Chen, Chenming Sun, Jinzhu Yin, Meng Zhang, Xiaogang Wu, Xuesong Li, Yonghong Wang

**Affiliations:** ^1^ College of Artificial Intelligence, Taiyuan University of Technology, Taiyuan, China; ^2^ Shanxi Bethune Hospital, Shanxi Academy of Medical Sciences, Tongji Shanxi Hospital, Third Hospital of Shanxi Medical University, Taiyuan, China; ^3^ Shanxi Provincial People’s Hospital, Taiyuan, China; ^4^ Central Laboratory of Sinopharm Tongmei General Hospital, Datong, China

**Keywords:** aneurysm, flow-diverting stents, hemodynamics, structure optimization, finite element analysis

## Abstract

Flow-diverting stents are crucial for aneurysm treatment, with their structural design significantly impacting post-implantation hemodynamics. While clinically effective, opportunities remain to enhance their flexibility, flow diversion capability, and long-term safety through ongoing structural optimization. In this study, with Pipeline Embolization Device (PED) as a reference, four kinds of flow-diverting stents with different braided cross-section shapes (quadrilateral, hexagon, octagon, and decagon) were designed under the condition of keeping the amount of material constant. Firstly, pure bending loads are applied to each stent through finite element analysis, and its flexibility is evaluated by analyzing the torque-angle curve. Secondly, the computational fluid dynamics method was utilized to simulate the hemodynamic characteristics after the implantation of each stent. The results show that: (1) Under the condition of bending 60°, the decagonal stent has the best flexibility, followed by the quadrilateral one. (2) The overall blood flow distribution of stents with different cross-sections is similar, but there are differences in the local average flow velocity of the tumor cavity: the circular one is the highest, and the quadrilateral one is the lowest. (3) The wall pressure gradient change of the polygonal stent is gentler than that of the circular one. Among them, the wall pressure of the hexagonal and decagonal stents is the maximum and the average pressure in the cavity is the lowest. (4) The area of the low WSS region on the aneurysm wall is the largest in quadrilaterals and the smallest in circles. On the maternal artery segment, the hexagon is the largest and the quadrilateral is the smallest. Comprehensive comparison shows that quadrilateral and decagonal cross-section stents exhibit better comprehensive performance. Through the above research, theoretical support can be provided for the optimal design of flow-diverting stents structures.

## 1 Introduction

An aneurysm is a lesion formed by abnormal local protrusion of the blood vessel wall. Its rupture may lead to subarachnoid hemorrhage and has a relatively high disability rate and mortality rate (Jiang et al., 2016; Xiang et al., 2025). Interventional therapy has gradually become the preferred treatment option for aneurysms due to its minimally invasive nature and high efficiency (Tawk et al., 2021; Nada et al., 2025). Flow-diverting stents, as a new generation of endovascular interventional devices, promote thrombosis at the neck of the aneurysm by changing the local hemodynamic environment, and ultimately achieve complete occlusion of the aneurysm cavity (Boite et al., 2023). Although flow-diverting stents have achieved certain results in clinical applications, they still face some challenges, such as the risk of thrombosis outside aneurysms, aneurysm rupture, and adaptability to complex anatomical structures. Therefore, further optimizing the stent design, expanding the scope of indications and improving safety remain the key directions of current research.

Flow-diverting stents play an important role in the treatment of vascular diseases, and their design directly affects the hemodynamic characteristics after implantation. Hemodynamic parameters such as flow velocity, pressure and wall shear stress (WSS) not only affect the long-term performance of the stent, but also are closely related to complications such as intimal hyperplasia of blood vessels and thrombosis. At present, a large number of studies have explored the hemodynamic characteristics of flow-diverting stents, but most of them have focused on a single cross-sectional shape, lacking systematic analysis of different cross-sectional shapes. Chao and Yue et al. (Wang et al., 2016; Zhang et al., 2019; Cillo-Velasco et al., 2020) investigated the influence of stent porosity on hemodynamics and mechanical properties of stents. The results showed that stents with low porosity reduced blood flow within aneurysms but increased stent rigidity, while stents with high porosity had better flexibility but poorer blood flow restriction effect. Ma et al. (2014) proposed a flow-diverting stent with a gradient pore structure. This stent is composed of a low-pore area in the middle and a high-pore area at the end. By rationally designing the structural parameters at the end of the stent, the gradient change of porosity from the middle to the end was achieved. The results show that this new type of stent shows great potential in the treatment of cerebral aneurysms. All the above-mentioned researchers used circular cross-section flow-diverting stent to explore the influence of stent porosity on regulating hemodynamics.

Jun et al. (2024) fabricated three different materials of flow-diverting stents under the same structure and systematically evaluated the differences in their physical properties. Zheng et al. (2019) conducted mechanical characterization on a new type of braided composite stent woven with nickel-titanium alloy wire and polyethylene terephthalate strips, and compared it with the nickel-titanium alloy flow-diverting stent of the same weaving method. The above-mentioned researchers mainly explored the influence of different material parameters on the mechanical properties of flow-diverting stents. Among them, the new type of composite material stent proposed by Zheng et al. is woven from different cross-sections (filamentous and strip-shaped), and ultimately the mechanical properties of the composite stent obtained are better. Nada et al. (2021) explored the influence of stent thickness and porosity on the hemodynamics within aneurysms. The results showed that the stent with the lowest thickness and porosity had the best performance and could promote the formation and healing of thrombi within aneurysms. Suzuki et al. (2017a) studied the relationship between the geometric parameters of the stent and its mechanical properties, and found that adjusting the support size can change the mechanical properties while minimizing the changes in porosity or pore density. Suzuki et al. (2017b) designed several stents by changing the thickness and weaving Angle of the stents for structural analysis. The results showed that changing the thickness and weaving Angle of the stents could adjust the mechanical properties while maintaining the same degree of flow reduction effect. Zhang et al. (2016) proposed a method for automatically optimizing the structure of flow-diverting stents. By rearranging the initial phase of the braided filaments, the porosity of the original device can be maintained. This method can be used to determine the braided filament arrangement structure with the optimal flow diversion efficiency. The above-mentioned researchers also used circular cross-section flow-diverting stents to explore the influence of some macroscopic parameters and weaving forms of the stents on the mechanical properties of the stents.

At present, the optimal design of flow-diverting stents mainly focuses on exploring the influences of macroscopic parameters such as stent porosity, braided strand number, braided Angle, and braided wire thickness, as well as stent materials and braided forms on hemodynamics, while the research on the microstructure characteristics of braided wires is relatively insufficient. It is worth noting that the differences in the geometric shape of the braided wire cross-section may significantly affect the mechanical properties and hemodynamic characteristics of the stent, thereby influencing the therapeutic effect and prognosis of the patient. Therefore, systematically studying the influence of the cross-sectional shape of braided wires on the performance of stents not only helps to optimize the design of existing flow-diverting stents, but also provides important theoretical basis and technical support for the development of a new generation of endovascular treatment devices.

With the rapid development of computing technology, Fluid-Structure Interaction (FSI) and Computational Fluid Dynamics (CFD) simulation have become important tools for studying the pathological characteristics of aneurysms (Wang et al., 2016; Masuda et al., 2022; Nada et al., 2022; Barahona et al., 2021; Uchiyama et al., 2021; Zhao et al., 2017). FSI can accurately capture the flow pattern changes near the wall surface and is an effective numerical tool for analyzing the interaction between fluids and solid structures, and while CFD is more suitable for studying the flow characteristics in the middle of the aneurysm cavity. Specifically, CFD has significant advantages in simulating the overall hemodynamics within the aneurysm cavity, while FSI is more suitable for analyzing the complex flow behavior near the aneurysm wall and its mechanical impact on the vascular wall. FSI usually requires higher computing resources and time costs, while CFD has higher computational efficiency and does not need to consider the deformation problem of fluid meshes, thereby being able to provide stable mesh quality. CFD has certain limitations in terms of energy conservation at the interface, while FSI can better meet this condition. At present, CFD has been widely used to study the influence of stent implantation on the hemodynamics of aneurysms (Nada et al., 2022; Li et al., 2018; Murayama et al., 2019). In clinical practice, hemodynamic studies of aneurysms often rely on medical image processing (e.g., CT/MRI segmentation) to reconstruct patient-specific vascular geometries. While such approaches provide anatomical accuracy, idealized models based on literature-derived parameters remain valuable for parametric analyses and mechanistic investigations. The choice between image-based and idealized models depends on research objectives: the former prioritizes individual relevance, whereas the latter enables controlled evaluation of geometric effects. In this study, an idealized aneurysm model was adopted, and the method of CFD was employed to investigate the effects of different cross-sectional stents on hemodynamics.

Based on this, this study referred to the widely used PED in clinical practice. The stent adopted a circular braided cross-section design, and its clinical efficacy and safety have been widely verified. In order to deeply explore the influence of the geometric shape of the braided wire cross-section on the performance of the stent, under the condition of maintaining a constant material dosage (consistent cross-sectional area of the braided wire), four different cross-sectional shapes (quadrilateral, hexagonal, octagonal, and decagonal) of flow-diverting stents were designed. Firstly, pure bending loads are applied to each stent through finite element analysis, and its flexibility is evaluated by analyzing the torque-angle curve. Secondly, the CFD simulation method was utilized to simulate the hemodynamic characteristics after the implantation of each stent, with a focus on analyzing the changes of key parameters such as the flow velocity field, pressure distribution, and WSS. Through the above systematic research, the aim is to clarify the correlation between the cross-sectional morphology of braided wires and flexibility as well as hemodynamic effects, providing theoretical support for the optimal design of stent structures.

## 2 Materials and methods

### 2.1 Stent structure

**FIGURE 1:**
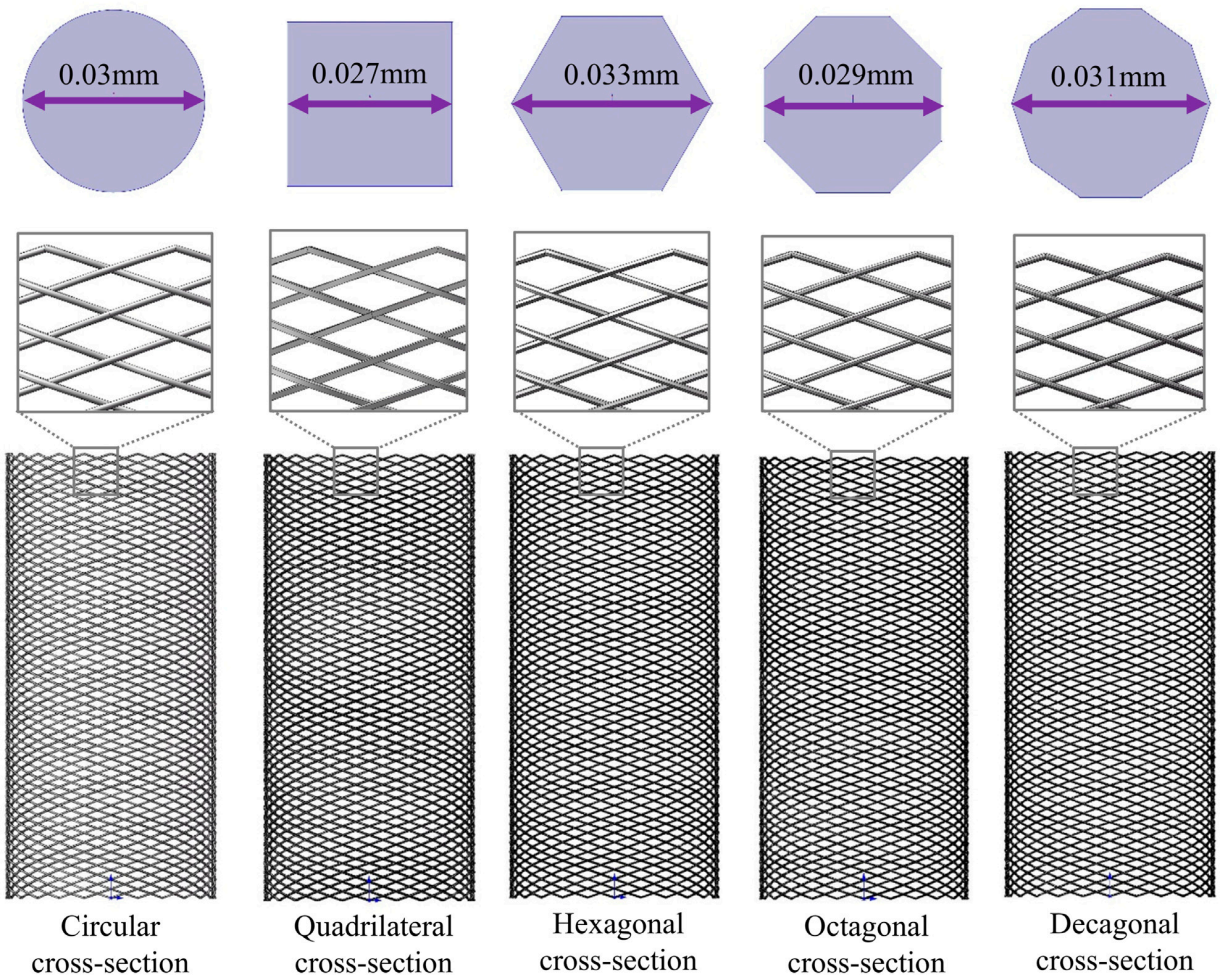
Three-dimensional models of five different cross-section stents and two-dimensional diagrams of different cross-sections.

The stent structure was based on PED, which is widely used in clinical practice. The PED adopts a circular cross-section design and is composed of 48 braided filaments with a diameter of 0.03 mm. The diameter of the stent is 4.75 mm, the length is 10 mm, and the porosity is approximately 70%. Its clinical efficacy and safety have been fully verified (Wang et al., 2016; Masuda et al., 2022; Maragkos et al., 2020). Based on the structural parameters of PED, maintaining a constant material usage, that is, keeping the cross-sectional area of the braided filament consistent (all 7.071 × 10^−4^ mm^2^), four different cross-sectional shapes (quadrilateral, hexagonal, octagonal, and decagonal) of flow-diverting stents were designed (Figure 1). The porosity measured successively was approximately 74.2%, 68.3%, 71.8%, and 71.5%, respectively.

### 2.2 Aneurysm model

In this study, cerebral artery diseases were taken as the research object, and medium-sized cystic aneurysms were selected to establish an idealized artery-aneurysm composite model (Figure 3a). The specific geometric parameters of the model are as follows: The length of the tumor-bearing arterial segment is 40 mm, its inner diameter is 4.75 mm, and its outer diameter is 5.75 mm. The inner diameter of the aneurysm was 9 mm, the outer diameter was 10 mm, and the height of the aneurysm was 7 mm. The thickness of the arterial wall is uniformly set at 0.5 mm (Cillo-Velasco et al., 2020; Masuda et al., 2022; Fan et al., 2024).

### 2.3 Analysis of bending flexibility

Nickel-titanium alloys are widely regarded as ideal materials for vascular stents due to their excellent superelasticity, shape memory effect, biocompatibility and mechanical properties, which can significantly improve the success rate and long-term prognosis of interventional therapy. In this study, the stent material was nickel-titanium alloy, with a density of 6.5 g/mm^3^ and a Poisson’s ratio of 0.35. The definition of material properties is implemented through the user subroutine (VUMAT) in the Abaqus software. For specific material parameters, please refer to Table 1 (Zheng et al., 2019). Through the verification of mesh sensitivity, the stents all adopt three-dimensional eight-node structural entity units for mesh division. The size of the surface mesh is 0.01 mm and the size of the volume mesh is 0.04 mm. The number of mesh nodes of the five stent models is 1052016, 940800, 865536, 900032, and 715008 respectively. The total number of units is 788004, 601344, 563760, 674163, and 526176 respectively.

**TABLE 1:** Material properties of the stent.

Nitinol	Material property value
Austenite elasticity E_A_	50000 MPa
Martensite elasticity E_M_	37000 MPa
Start of transformation loading $\sigma^{S\;L}$	400 MPa
End of transformation loading $\sigma^{E\;L}$	650 MPa
Start of transformation unloading $\sigma_{U}^{S}$	350 MPa
End of transformation unloading $\sigma_{U}^{E}$	80 MPa
Volumetric transformation strain $\varepsilon_{V}^{L}$	0.055

**FIGURE 2:**
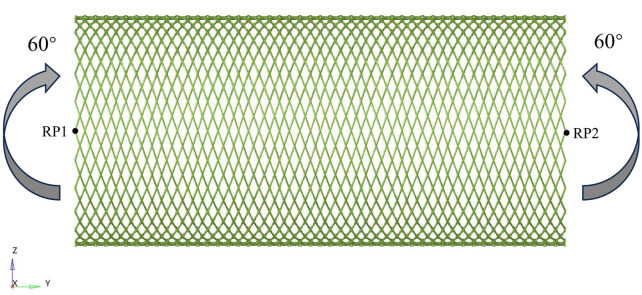
Schematic diagram of the finite element model for testing the flexibility of the stent.

When evaluating the bending flexibility of the stent, the mechanical test of the stent was conducted by using the pure bending loading method. The established finite element model is shown in Figure 2. The boundary conditions of the stent model were carefully defined to accurately simulate its mechanical behavior during bending deformation. Reference points RP1 and RP2 are respectively set at both ends of the Z-axis direction of the stent, and the beam element connection technology is used to establish an association with the terminal node through multi-point constraint (MPC). The main purpose was to constrain the displacement of the nodes along the axial direction of the beam, while allowing their lateral free movement. This constraint condition ensures that the cross-sections at both ends of the stent remain circular in shape during the bending test, thereby more accurately simulating the mechanical behavior under actual working conditions and improving the reliability of the analysis results. Angular displacement loads around the X-axis are applied at the preset nodes, and the bending angle gradually increases to 60°. The flexibility of the stent is quantified by monitoring and recording the corresponding bending moment load curves. Considering the friction effect at the intersection of the stents, the contact type is set as universal contact and the friction coefficient is set to 0.3 (Zheng et al., 2019; Kim et al., 2008; Zaccaria et al., 2020). Based on the advantages of the ABAQUS/Explicit solver in complex contact simulation, this solver was selected for numerical calculation in this study. When conducting quasi-static simulation, strictly control the ratio of the kinetic energy to the internal energy of the stent to ensure that the kinetic energy is maintained below 5% of the total internal energy, thereby effectively avoiding numerical calculation errors caused by inertial forces.

### 2.4 Hemodynamic analyses

**FIGURE 3:**
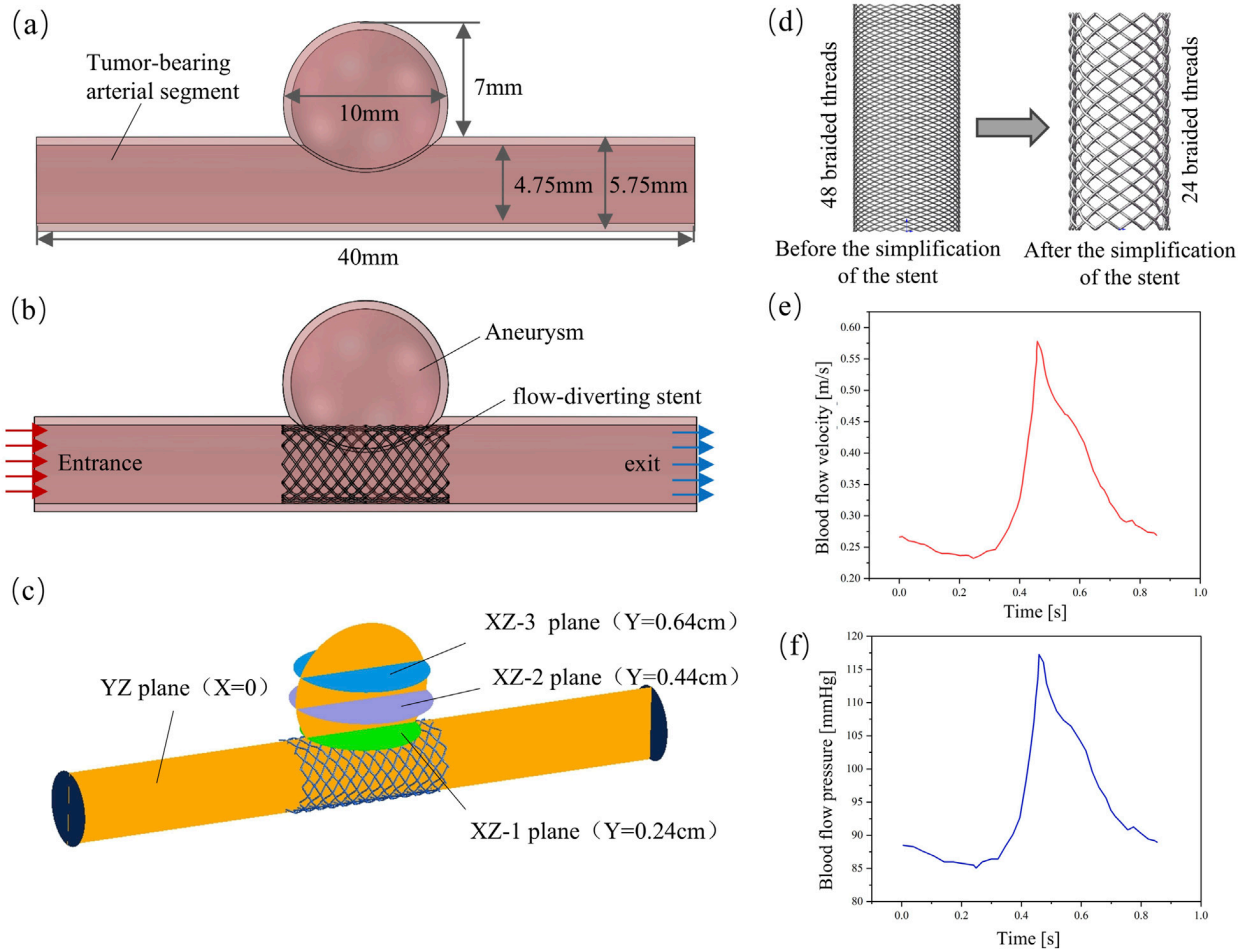
Finite element model of hemodynamic analysis; **(a)** Aneurysm model; **(b)** Solid-domain model; **(c)** Evaluate the planar position of blood flow parameters in the aneurysm cavity; **(d)** Simplified stent model; **(e)** Entry boundary conditions; **(f)** Exit boundary conditions.

A solid domain model integrating the stent-bearing artery segment was constructed (Figure 3b), and the fluid domain model was extracted through the Boolean operation method. This paper simplifies the stent, and the model is shown in Figure 3d. The main reason is that the braided filament diameter of the stent is small and the number of strands is large, which will lead to an excessive number of meshes when meshing the fluid domain in hemodynamic analysis. Due to the limitations of the calculation conditions, we made appropriate adjustments to the size of the stent: the diameter of the braided wire was expanded to 0.1 mm, the number of braided strands was reduced to 24 strands, and other parameters remained unchanged. It should be noted that this size adjustment is only used to simplify the calculation and will not affect the final result of the hemodynamic impact of stents with different cross-sections. The hemodynamic characteristics of each stent after implantation were estimated by the CFD method using ANSYS Fluent 2022R1 (ANSYS, US). To generate the numerical mesh, the volume model was discretized using the Ansys Fluent Mesher module. The mesh generation method based on polyhedral elements is adopted. Meanwhile, three layers of expansion are generated in the wall area to obtain more accurate results near the wall and the stent unit (Reorowicz et al., 2022). To ensure that the calculation results are not affected by the mesh density, the mesh independence is verified based on WSS and the velocity distribution within the aneurysm. When the WSS and flow velocity distribution are within the 2.5% error range of the finest mesh, it is considered to have reached the convergence criterion (Nada et al., 2022). Convergence was achieved when the minimum size of the unit in the support area of the stent was controlled at 0.01 mm and the overall unit size was 0.15 mm. Eventually, the total number of mesh elements in the fluid domain model obtained was between 1 million and 3 million.

When conducting CFD numerical simulation, considering the flow characteristics of blood, the use of transient analysis can better simulate the blood flow characteristics under physiological conditions. The blood was simplified to a homogeneous and incompressible Newtonian fluid, with its density set at 1,060 kg/m^3^ and dynamic viscosity at 0.0035 Pa•s. The Reynolds number Re calculated based on these fluid property parameters is significantly lower than 2,000. According to the theory of fluid mechanics, when Re is less than 2,000, viscous force dominates, the flow presents stable laminar flow characteristics, and the turbulent effect can be ignored. Therefore, it is reasonable to use the laminar flow model for calculation (Wei et al., 2019). Suppose the arterial wall and stent are rigid. To accurately simulate the blood flow environment of the intracranial vascular system, the boundary conditions were physiologically optimized. At the entrance of the fluid domain, a blood flow velocity waveform within a complete cardiac cycle was applied, as shown in Figure 3e. The exit boundary conditions are defined using the pressure waveforms within a complete cardiac cycle, as shown in Figure 3f, to more truly reflect the blood flow characteristics under physiological conditions (Barahona et al., 2021). When setting the conditions, the calculation model is first subjected to standard initialization processing. The initial conditions are as follows: the pressure field values throughout the computational domain are all 0 Pa, and in the velocity field, only the z-axis direction has an initial velocity of 0.27 m/s, while the initial velocities in the x-axis and y-axis directions are both 0 m/s. In hemodynamic studies, the changes of parameters such as flow velocity, pressure and WSS are often used to establish the association between hemodynamic characteristics and clinical outcomes (Suzuki et al., 2017b; Sindeev et al., 2018). However, in order to analyze the hemodynamic characteristics of different regions within the aneurysm cavity more systematically, in this study, three parallel planes and one vertical plane were constructed within the aneurysm cavity. The vertical plane was named the “YZ plane” according to the three-dimensional space where it was located. The three parallel planes were respectively named “XZ-1 plane”, “XZ-2 plane” and “XZ-3 plane” according to the three-dimensional space they were in and in the order of the smallest distance from the mother artery. The specific location is shown in Figure 3c.

## 3 Results

### 3.1 Bending flexibility

**FIGURE 4:**
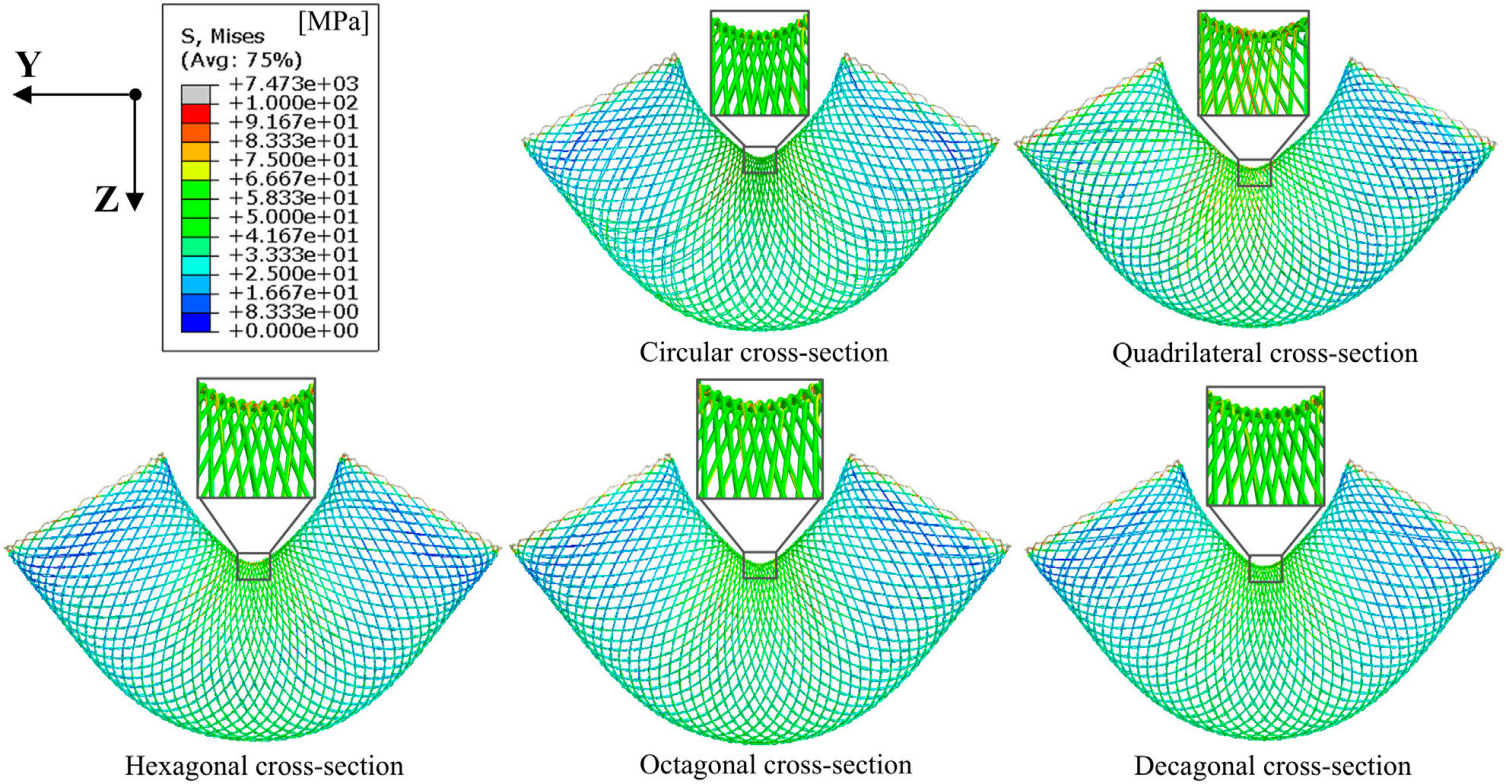
Stress distribution cloud diagrams of stents with different cross-sections when the bending angle is 60°.

**FIGURE 5:**
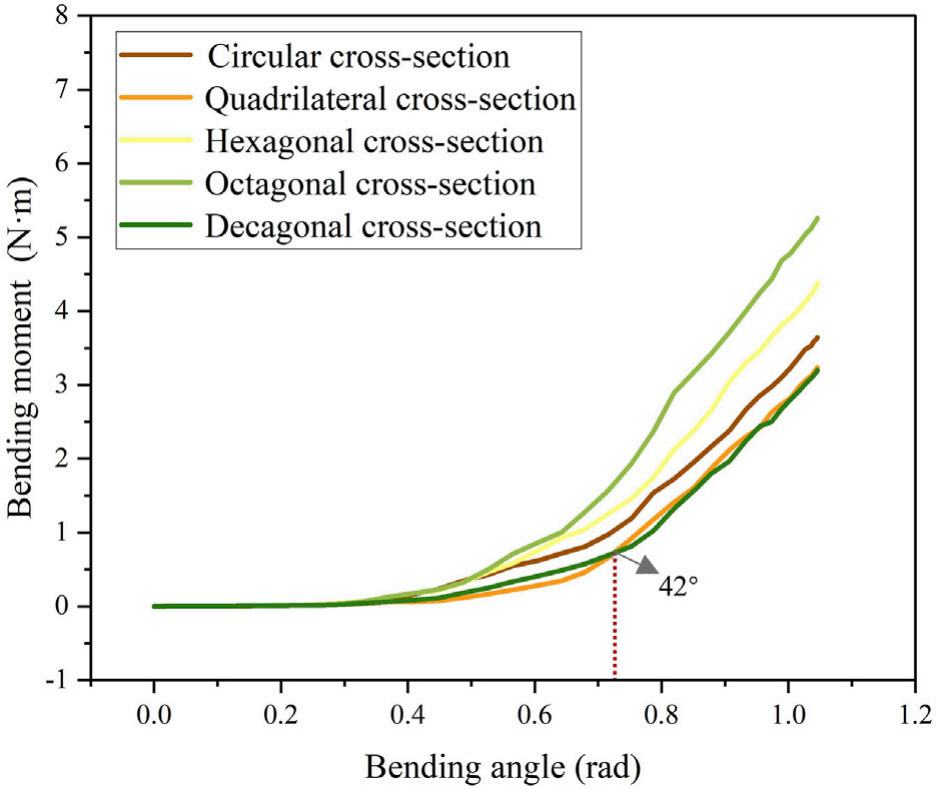
Comparison of bending performance of stents with different cross-sections.

The stress cloud diagram of the stent under the action of bending moment is shown in Figure 4. When the stent bends, the porosity in the bending area of the stent gradually decreases, and the constraint between the braided wires increases. Therefore, the stress value in the bending area of the stent is relatively large. From the local magnified images of the bending areas of each stent in Figure 4, it can be seen that the stress distribution on the stents with circular and decagonal cross-sections is relatively uniform, while the stress distribution on the other stents is relatively uneven, with greater stress in the geometric corner areas. The bending moment and rotation angle curves of the stent in the bent state are shown in Figure 5. When the stent is bent by 60°, the corresponding bending moments of the five stents are 3.6 N/m, 3.23 N/m, 4.4 N/m, 5.3 N/m and 3.2 N/m respectively. Bending moment is negatively correlated with flexibility. Under the same bending angle conditions, the greater the measured bending moment, the worse the flexibility. Therefore, under the condition of bending 60°, the flexibility of the flow-diverting stent with a circular cross-section is better than that of the stents with hexagonal and octagonal cross-sections, but not as good as that of the stents with quadrilateral and decagonal cross-sections. From the bending moment and angle curves of each stent in Figure 5, it can be obtained that the bending performance of the quadrilateral section and the decagonal section is generally comparable. When the bending angle is less than 42°, the bending moment of the decagonal section is slightly greater than that of the quadrilateral section. When the bending angle is greater than 42°, the bending moment of the decagonal section is slightly less than that of the quadrilateral section. Therefore, when implanting stents for arteries with small curvatures, quadrilateral cross-section flow-diverting stents perform better. When implanting stents for arteries with large curvatures, decagonal cross-section flow-diverting stents perform better.

### 3.2 The variation of flow velocity within the arterial segment

**FIGURE 6:**
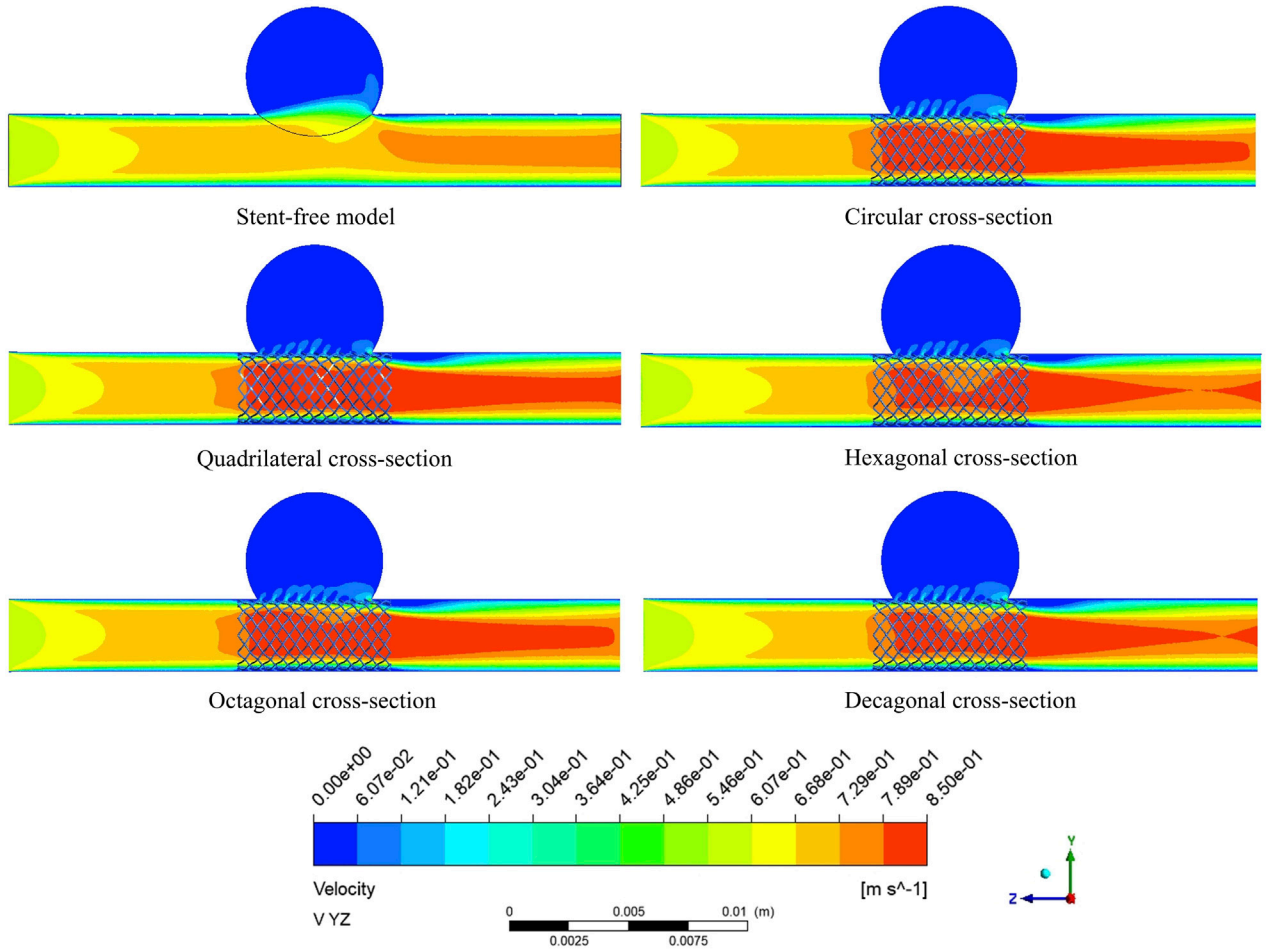
The velocity distribution cloud map on the YZ plane at the peak.

The velocity cloud graph on the YZ plane at the peak systolic moment (0.46 s) was selected for analysis (Figure 6). After stent implantation, the blood was significantly blocked from entering the aneurysm cavity. The distribution characteristics of the blood flow velocity field in the five models containing stents are similar. Therefore, it can be indicated that stents of different cross-sections do not affect the overall distribution of blood. The maximum flow velocity on the YZ plane was quantitatively evaluated. As shown in Figure 8a, the maximum flow velocities of the six models in the YZ plane were 79.1 cm/s, 93 cm/s, 90.5 cm/s, 87.8 cm/s, 90.8 cm/s, and 89.3 cm/s respectively. It can be obtained that after stent implantation, the flow velocity of the mother artery was increased and maintained within the normal physiological range.

**FIGURE 7:**
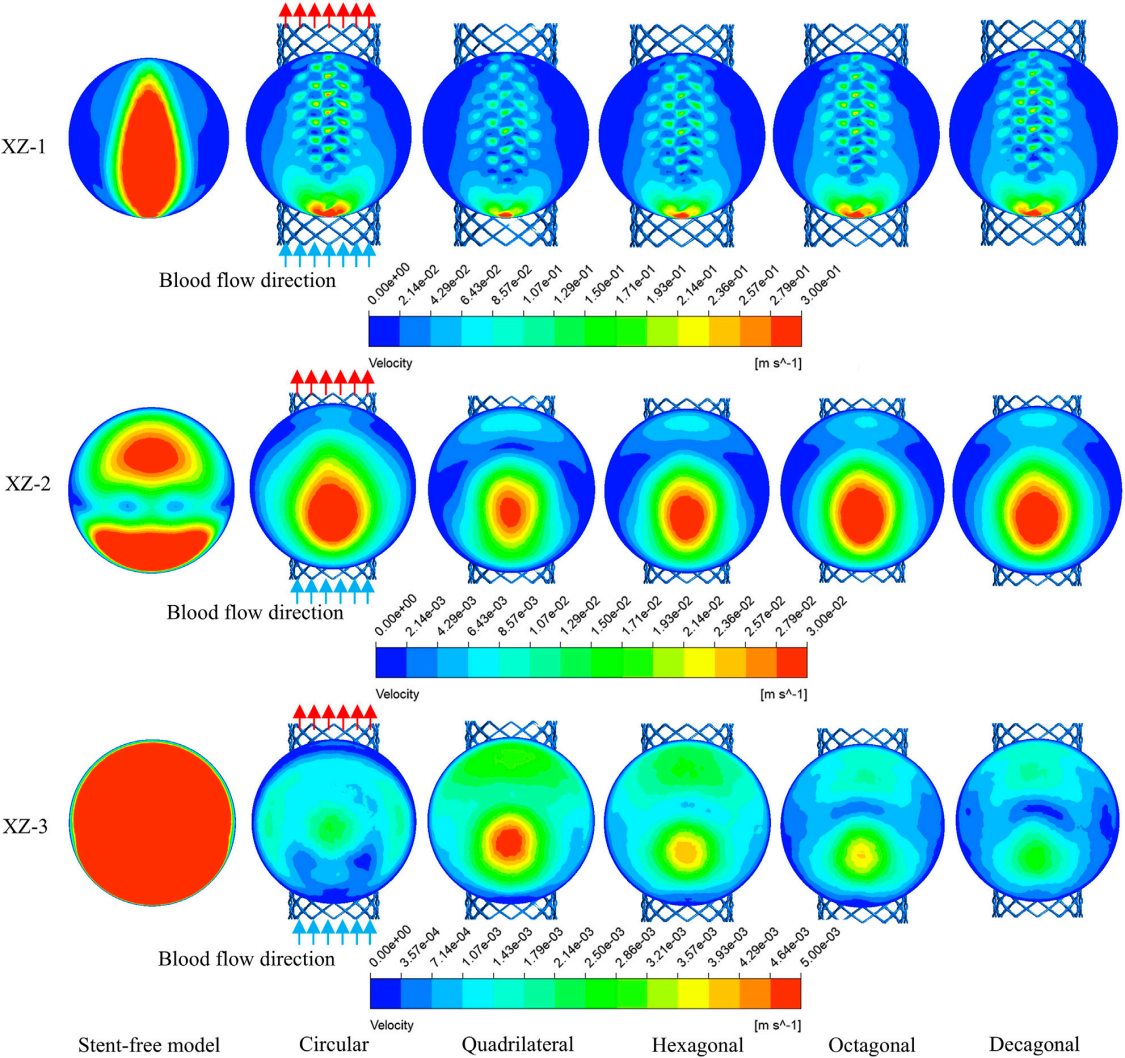
The velocity distribution cloud maps on the XZ-1, XZ-2, and XZ-3 planes at the peak.

**FIGURE 8:**
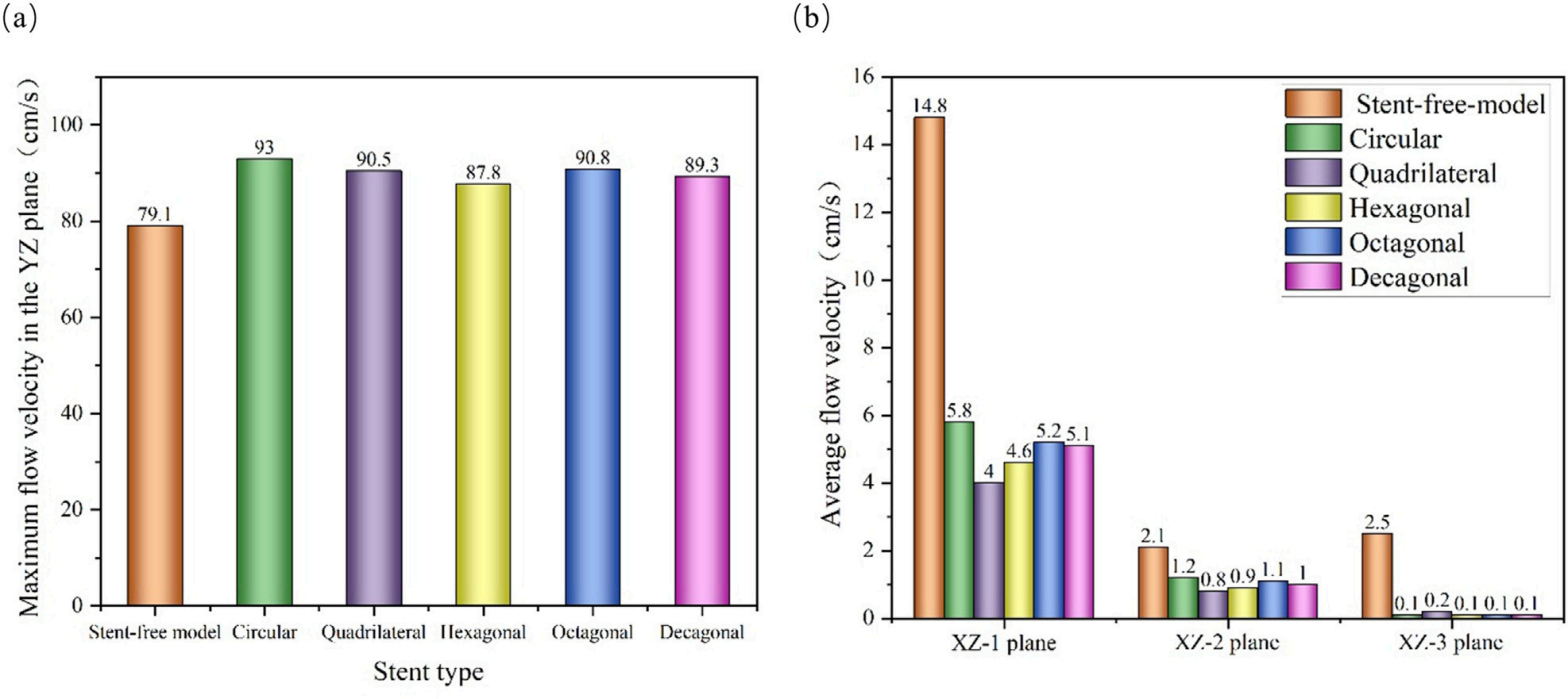
Flow velocity distribution characteristics of different cross-sections; **(a)** The maximum flow velocity on the YZ plane; **(b)** The average flow velocity on the XZ-1, XZ-2 and XZ-3 planes.

The flow velocity changes in the aneurysm cavity after stent implantation were analyzed by cutting the flow velocity cloud diagrams on the three planes within the aneurysm cavity. It can be seen from Figure 7 that the flow velocity in the aneurysm cavity was significantly reduced after stent implantation. The flow velocities on the XZ-1, XZ-2 and XZ-3 planes decreased successively, and the flow velocity distributions of the five stents on each plane were similar. The average flow velocities of each model on the three planes were quantitatively evaluated. As shown in Figure 8b, the average flow velocities of the six models on the XZ-1 plane were 14.8 cm/s, 5.8 cm/s, 4 cm/s, 4.6 cm/s, 5.2 cm/s, and 5.1 cm/s, respectively. The average flow velocities on the XZ-2 plane were 2.1 cm/s, 1.2 cm/s, 0.8 cm/s, 0.9 cm/s, 1.1 cm/s and 1 cm/s respectively. The average flow velocities on the XZ-3 plane were 2.5 cm/s, 0.1 cm/s, 0.2 cm/s, 0.1 cm/s, 0.1 cm/s and 0.1 cm/s respectively. It can be obtained from the average flow velocities calculated on the three planes that on the XZ-1 plane closest to the neck of the aneurysm and the XZ-2 plane in the middle of the aneurysm, the average flow velocity of the polygonal cross-section stent is smaller than that of the circular cross-section stent, which can better block the blood flow into the aneurysm cavity. While on the XZ-3 plane at the top of the aneurysm, the average flow velocity of the quadrilateral cross-section stent is slightly larger. The implantation of the stent has a greater impact on the flow velocity in the area close to the aneurysm neck, but a smaller impact on the area at the top of the distal aneurysm.

### 3.3 Pressure changes within arterial segments

**FIGURE 9:**
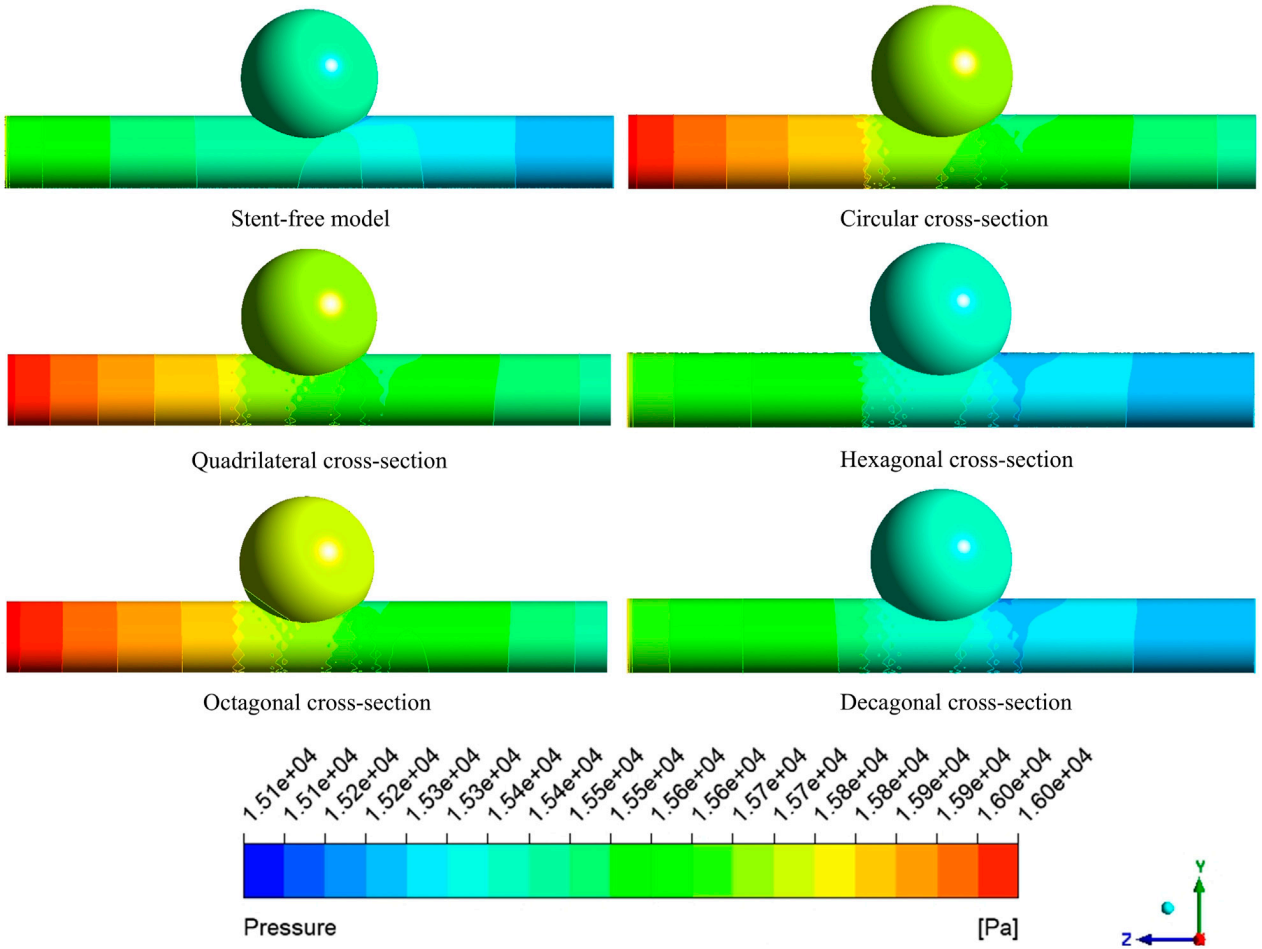
The pressure distribution cloud map on the arterial wall at the peak.

The pressure cloud diagrams on the wall at the peak time of the contraction period (0.46 s) were selected for analysis (Figure 9). The overall distribution of the pressure cloud diagrams of the six models was similar. The maximum stress was mainly distributed at the entrance of the model, and the pressure gradually decreased from left to right along the axial direction. The blood pressure distribution ranges of the six models were 15.14 kPa–15.79 kPa, 15.29 kPa–16.19 kPa, 15.41 kPa–16.19 kPa, 15.13 kPa–15.84 kPa, and 15.37 kPa-16.20 in sequence kPa, 15.09 kPa–15.84 kPa. From the perspective of distribution range, the blood pressure value of the stent-free model was smaller compared with that of the stent-containing model. In the stent-containing model, the pressure change gradient on the polygonal cross-section stent wall was smaller than that on the circular cross-section stent wall. No sudden pressure change occurred in all stent models. The maximum pressure on the wall surface was quantitatively evaluated, as shown in Figure 11a. Numerically, the maximum blood pressure of the stent-free model was the smallest, the maximum blood pressure of the circular and quadrilateral cross-section stents was similar, the maximum blood pressure of the hexagonal and decagonal cross-section stents was similar, and the maximum blood pressure of the octagonal cross-section stent was the largest.

**FIGURE 10:**
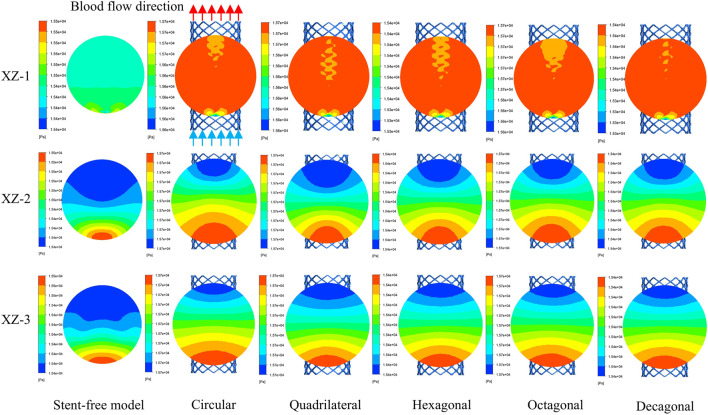
The pressure distribution cloud diagrams on the XZ-1, XZ-2, and XZ-3 planes at the peak.

**FIGURE 11:**
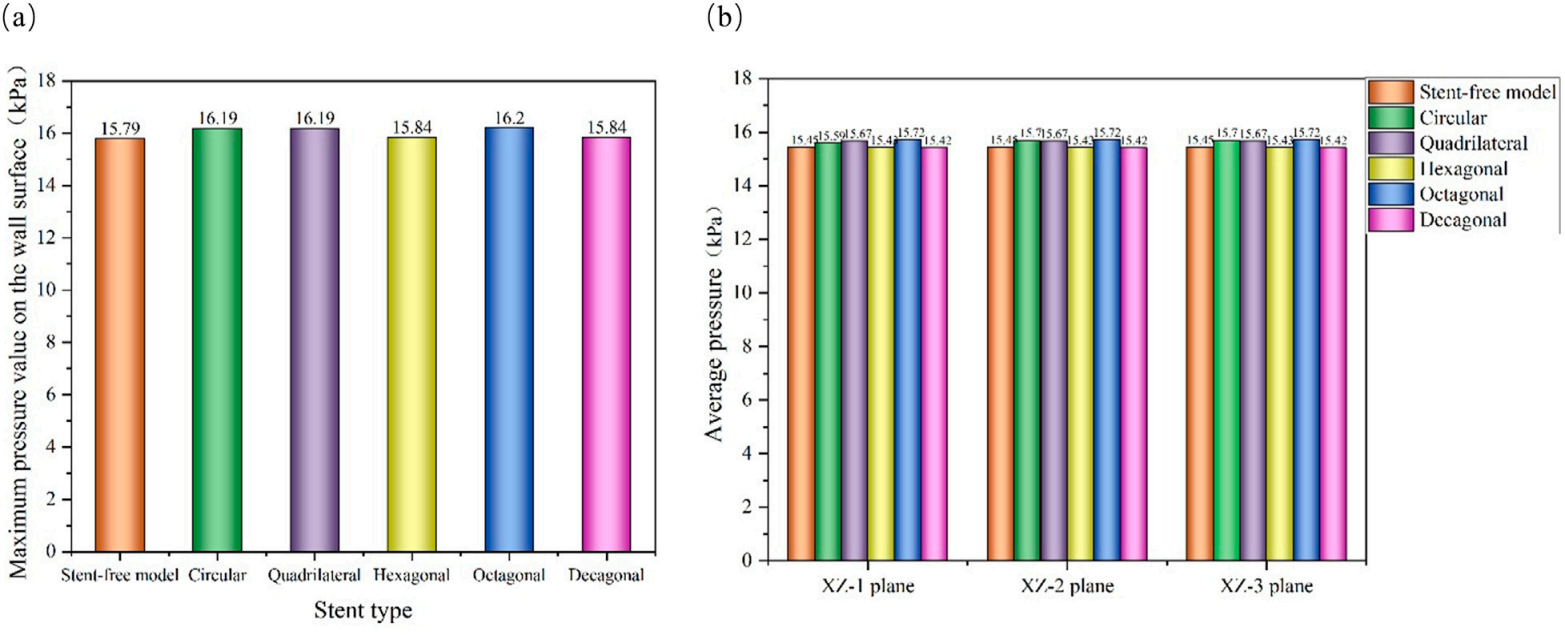
Pressure distribution characteristics at the peak; **(a)** The maximum wall pressure value at the peak; **(b)** The average pressure on the XZ-1, XZ-2, and XZ-3 planes at the peak.

The pressure changes in the aneurysm cavity were analyzed by extracting the pressure cloud maps on three planes within the aneurysm cavity (Figure 10), and the pressure distribution on each plane after the implantation of the five stents was similar. On the XZ-1 plane, the pressure distribution of the model without the stent is relatively uniform, while for the model with the stent, in the area with lower pressure, it is mainly concentrated in the area below the plane and near the stent pore area above. On the XZ-2 and XZ-3 planes, the blood flow pressure distributions of the six models show similar patterns, all showing a gradient decreasing trend of pressure along the blood flow direction. The results of quantitatively evaluating the average pressures of each model on the three planes are shown in Figure 11b. The average pressures of the six models on the XZ-1 plane are 15.45 kPa, 15.69 kPa, 15.67 kPa, 15.43 kPa, 15.72 kPa, and 15.42 kPa, respectively. The average pressures on the XZ-2 plane were 15.45 kPa, 15.7 kPa, 15.67 kPa, 15.43 kPa, 15.72 kPa and 15.42 kPa respectively. The average pressures on the XZ-3 plane were 15.45 kPa, 15.7 kPa, 15.67 kPa, 15.43 kPa, 15.72 kPa and 15.42 kPa respectively. Data show that there is almost no difference in the average pressure on the three planes. The average pressure of the hexagonal and decagonal cross-section stents on the three planes is the smallest, which can reduce the continuous damage to the vascular wall and lower the possibility of aneurysm rupture.

### 3.4 The variation of WSS

**FIGURE 12:**
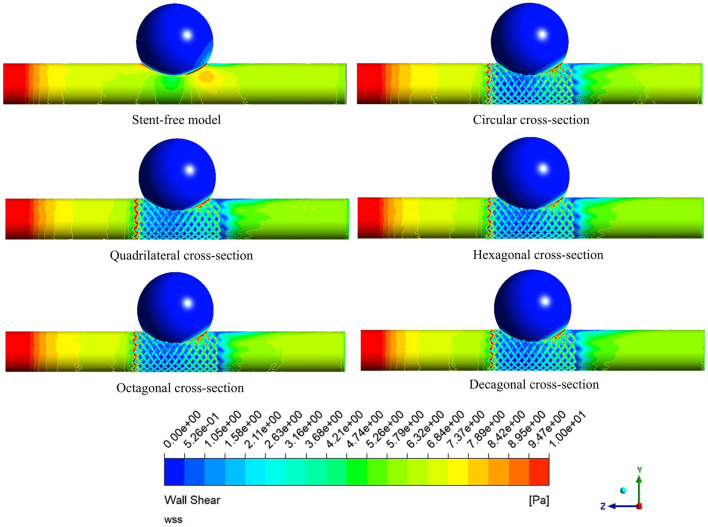
Cloud map of WSS distribution on the arterial wall at the peak.

**FIGURE 13:**
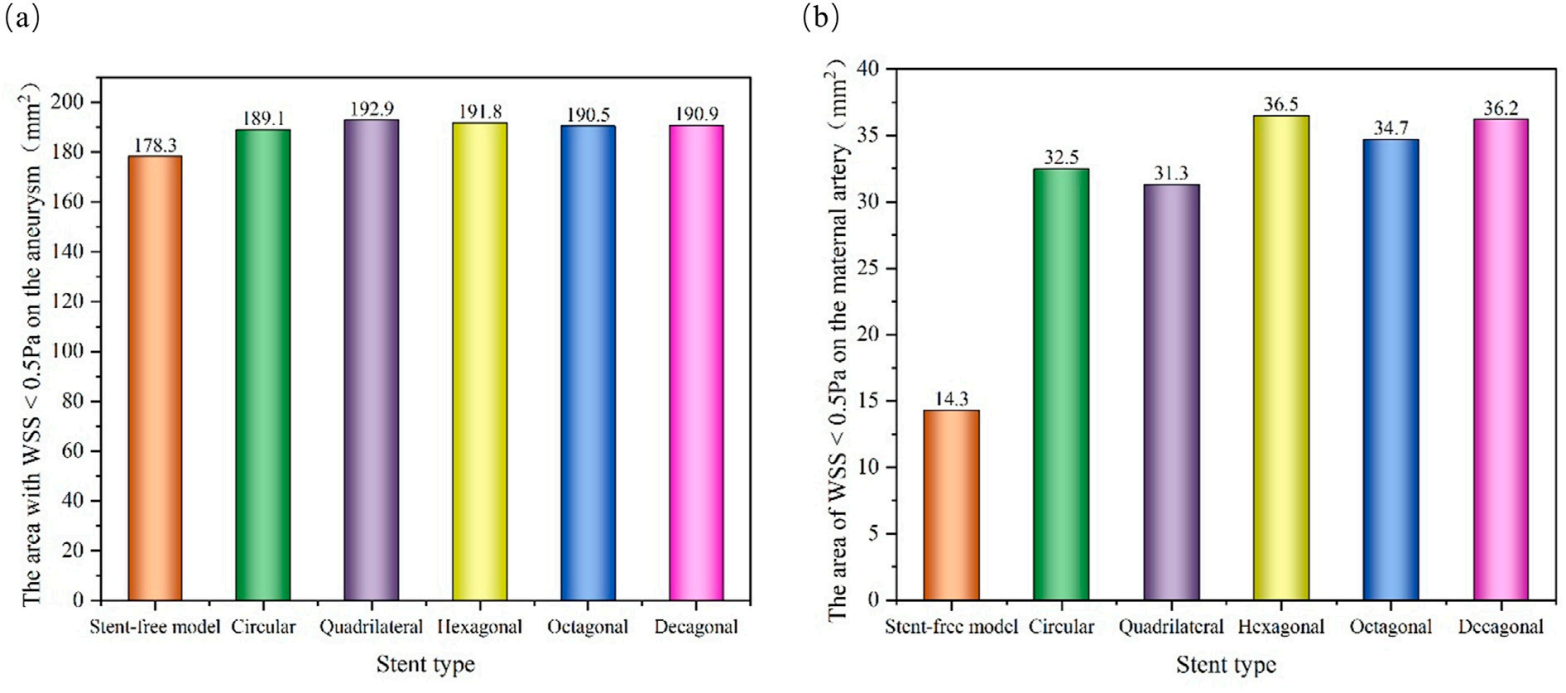
Distribution of low WSS areas at peak; **(a)** The area of low WSS (<0.5 Pa) on the aneurysm at the peak; **(b)** The area of low WSS (<0.5 Pa) on the maternal artery at the peak.

The WSS cloud maps at the peak systolic moment (0.46 s) were selected for analysis (Figure 12). The high WSS distribution of the six models was similar, mainly distributed at the vascular inlet, which was mainly caused by the higher inlet flow velocity. The low WSS distribution was slightly different. In the stent-free model, the low WSS was mainly distributed on the aneurysms. The low WSS of the model with stents was mainly distributed in the aneurysm and stent pore areas. Since low WSS (<0.5 Pa) is related to cell proliferation, intimal thickening and inflammation, thrombosis is more likely to form. If a large amount of low WSS is distributed on the aneurysm, it is more conducive to the occlusion of the aneurysm. If there is a considerable amount of low WSS near the stent in the maternal artery segment, it will be more likely to cause restenosis within the stent. Therefore, we quantitatively analyzed the areas of low WSS on aneurysms and maternal arteries in each model (Figure 13). The areas of low WSS on aneurysms in the six models were 178.3 mm^2^, 189.1 mm^2^, 192.9 mm^2^, 191.8 mm^2^, 190.5 mm^2^, and 190.9 mm^2^, respectively. The areas of low WSS on the maternal artery were 14.3 mm^2^, 32.5 mm^2^, 31.3 mm^2^, 36.5 mm^2^, 34.7 mm^2^, and 36.2 mm^2^ respectively. It can be obtained from the results that on the aneurysm, since the stent blocks the blood flow entering the aneurysm and reduces the impact of the blood flow on the aneurysm wall, the low WSS area of the model with the stent is higher than that of the model without the stent, which also indicates that stent implantation is more conducive to the occlusion of the aneurysm. Among the five stent models, the polygonal cross-section stent has a better effect than the circular cross-section stent, among which the quadrilateral cross-section stent is the best. On the maternal artery segment, since the stent protrudes into the normal artery segment, it will cause turbulence near the stent, resulting in more low WSS areas. Therefore, compared with the stent-free model, the low WSS area of the stent-containing model is higher, among which the low WSS area of the quadrilateral cross-section stent is the least.

## 4 Discussion

This study focuses on the influence of the geometry of the braided section on the bending flexibility and hemodynamic parameters of the stents in the design of flow-diverting stents. The flexibility of the stent is of vital importance as it determines whether the stent can well adapt to the complex anatomical structure of the blood vessel (such as changes in curvature and diameter). Insufficient flexibility may lead to fatigue fracture or displacement of the stent, affecting the therapeutic effect (Zheng et al., 2019). Meanwhile, the change in the local blood flow pattern after stent implantation is the key to promoting intratumoral thrombosis, but it may also bring the risk of distal vascular interference or thrombosis (Brinjikji et al., 2013; Zhao et al., 2023). Therefore, optimizing the stent design must take into account both good flexibility and favorable hemodynamic characteristics. This paper explores how different braided cross-sectional shapes affect the above two core performance parameters, aiming to provide a theoretical basis for optimizing the design of flow-diverting stents.

When evaluating the influence of braided cross-sections on stent flexibility, results reveal a nonlinear relationship between flexibility and cross-sectional morphology. At 60° bending, stents with different cross-sections show significant flexibility differences: circular cross-sections outperform hexagonal and octagonal designs but are less flexible than quadrilateral and decagonal ones. The quadrilateral and decagonal stents exhibit similar overall bending performance, though with subtle variations across different bending angles. This discovery indicates that the cross-sectional geometry of the braided wire has a significant influence on the flexibility of the flow-diverting stents, and the degree of influence is related to the range of bending angles. This conclusion is related to the research results of Zheng et al. (2019). They found that when stents were implanted in small-curvature arteries, the flexibility of the polyester tape - nickel-titanium alloy wire composite stent was significantly better than that of the nickel-titanium alloy wire braided structure, and the cross-sections of the braided wires of the two stents were different. This also indicates that by optimizing the structural design of the braided filament, the flexibility of the stent can be significantly changed, and its mechanical response is closely related to the bending angle.

High-speed blood flow and high WSS can inhibit thrombosis in the aneurysm lumen, while stent implantation can promote thrombosis by reducing blood flow velocity and WSS. Maintaining a relatively high blood flow velocity in the maternal artery helps keep the tumor-bearing vessels unobstructed, reduces the risk of intimal hyperplasia, and thereby avoids vascular restenosis at the stent implantation site caused by intimal hyperplasia (Mut et al., 2010). Through hemodynamic analysis, it was found that the implantation of flow-diverting stents with different cross-sections could significantly reduce the blood flow velocity in the aneurysm lumen and simultaneously increase the maximum velocity of the mother artery. This finding is consistent with the research results of Shunsuke et al., that is, regardless of the size of the aneurysm, the blood flow velocity and volumetric velocity within the aneurysm decrease with the reduction of stent porosity (Masuda et al., 2022). This study shows that compared with the stent-free model, the five stent models all exhibited the characteristic of reduced flow velocity within aneurysms due to the significant reduction in porosity. However, since the porosity differences of the five stents adopted in this study were relatively small, while the different porosities studied by Shunsuke et al. varied greatly. Therefore, the results obtained in this paper do not fully conform to the linear relationship between porosity and flow velocity proposed by Masuda et al. (2022). The blood flow guidance effect of the polygonal cross-section in the aneurysm neck region is better than that of the circular cross-section, but the difference is not significant in the top region of the aneurysm. This might be due to the angular structure of the polygonal cross-section stent increasing local turbulence or micro-vortices, further hindering the blood flow into the aneurysm cavity. However, the blood flow in the top area of the aneurysm has significantly attenuated, and the influence of the cross-sectional shape has weakened.

Elevated intra-aneurysmal pressure increases wall stress, potentially exceeding tissue tolerance and causing rupture. Rapid pressure fluctuations additionally induce damaging hemodynamic impacts on vessel walls. Minimizing blood pressure gradients is therefore critical for reducing vascular wall injury (Zhang et al., 2021). Compared with circular cross-section stents, quadrilateral, hexagonal and decagonal cross-section stents can improve the gradient change of blood pressure on the wall surface, making the pressure distribution on the wall surface more stable. Thus, they will not have a significant impact on the vascular wall and bear a smaller maximum pressure on the vascular wall. The research of Wang et al. suggests that after stent implantation, the aneurysm shows the characteristics of decreased flow velocity and increased pressure, which is in line with the basic law of Bernoulli’s principle (Wang et al., 2016). It is worth noting that although the structural differences among the five different cross-section stents are relatively small, the angular regions of the polygonal cross-section can still induce local flow separation and vortex generation. However, the influence of this local flow disturbance on the overall pressure distribution is relatively limited, and the pressure differences among stents with different cross-sections have not reached a significant level.

The low WSS area on the aneurysm wall is positively correlated with aneurysm occlusion. An increase in the low WSS area is conducive to thrombosis and aneurysm lumen occlusion within the aneurysm (Bernardini et al., 2021). However, in the maternal artery segment, the low WSS area near the stent may increase the risk of in-stent restenosis. Quantitative analysis shows that compared with the circular cross-section stent, the polygonal cross-section stent can induce a larger low WSS area on the aneurysm wall, among which the quadrilateral cross-section stent performs the best. Furthermore, on the wall of the maternal artery, the coverage area of the low WSS region of the quadrilateral cross-section stent is the smallest, indicating that it may reduce the occurrence probability of restenosis within the stent. This finding is partially consistent with the research conclusion of Mut et al. (2010), that is, the rectangular (length-to-width ratio of 1) pillar stent is superior to other cross-sections in promoting aneurysm thrombosis. However, in the study by Yong et al., a complete stent model was not adopted. Instead, the shape of the annular stent pillar was used for exploration, and the single index of blood flow reduction rate was used as the evaluation index of stent efficacy (Mut et al., 2010). This study expanded the optimization range of the cross-sectional shape of the stent and adopted a braided stent model that was closer to the real situation, providing a more reliable basis for clinical stent design.

This study also has some limitations. First of all, the aneurysm model adopted is idealized. Secondly, the numerical simulation is based on the rigid wall hypothesis and does not take into account the interaction between blood flow and the arterial wall, the real mechanical behavior of the stent, and arterial compliance. This may affect the flow pattern and thereby influence related parameters such as WSS. Furthermore, although the stent implantation simulation achieved the ideal state of complete adhesion, the possible poor adhesion of the stent in actual clinical operations was not considered. Finally, the research results have not yet been verified through *in vitro* experiments or clinical follow-up data, which may affect the clinical applicability of the conclusion.

## 5 Conclusion

In this study, under the condition of constant material dosage, the comprehensive performance of flow-diverting stents with different cross-sectional shapes (circular, quadrilateral, hexagonal, octagonal, and decagonal) was compared and analyzed. Finally, the comprehensive performance of the flow-diverting stents with quadrilateral and decagonal cross-sections is better, providing theoretical support for the optimal design of the flow-diverting stents structure. The main conclusions are summarized as follows:

1. The quadrilateral and decagonal cross-section stents demonstrate the best overall bending flexibility.
2. Stents of different cross-sections have a relatively small impact on the overall blood flow distribution. In the aneurysm neck and middle region of the aneurysm, the polygonal cross-section stent can more effectively reduce the average flow velocity in the aneurysm lumen than the circular cross-section stent. In the area at the top of the tumor, the average flow velocities of each stent were not significantly different.
3. The maximum pressure on the wall of the hexagonal and decagonal cross-section stents and the average pressure value within the tumor cavity are relatively low.
4. Quadrilateral cross-section stents have the best effect in expanding the low WSS area on the aneurysm wall, followed by other polygonal stents, and circular stents are relatively poor. All stent implants would increase the low WSS area on the maternal artery segment, but the negative impact of the quadrilateral cross-section stent was the least.

## Data Availability

The original contributions presented in the study are included in the article/supplementary material, further inquiries can be directed to the corresponding authors.

## References

[B1] Barahona J. Valencia A. Torres M. (2021). Study of the hemodynamics effects of an isolated systolic hypertension (ISH) condition on cerebral aneurysms models, using FSI simulations. Appl. Sci. 11 (6), 2595. 10.3390/app11062595

[B2] Bernardini A. Larrabide I. Morales H. G. Pennati G. Petrini L. Cito S. (2021). Influence of different computational approaches for stent deployment on cerebral aneurysm haemodynamics. Interface Focus 1 (3), 338–348. 10.1098/rsfs.2011.0004 22670204 PMC3262449

[B3] Boite Y. Klein T. S. Medronho R. A. Wajnberg E. (2023). Numerical simulation of flow-diverting stent: comparison between branches in bifurcation brain aneurysm. Biomech. Model Mechanobiol. 22 (6), 1801–1814. 10.1007/s10237-023-01733-2 37335373

[B4] Brinjikji W. Murad M. H. Lanzino G. Cloft H. J. Kallmes D. F. (2013). Endovascular treatment of intracranial aneurysms with flow diverters: a meta-analysis. Stroke 44 (2), 442–447. 10.1161/strokeaha.112.678151 23321438

[B5] Cillo-Velasco P. R. Luciano R. D. Kelly M. E. Peeling L. Bergstrom D. J. Chen X. B. (2020). The hemodynamics of aneurysms treated with flow-diverting stents considering both stent and aneurysm/artery geometries. Appl. Sci. 10 (15), 5239. 10.3390/app10155239

[B6] Fan X. Z. Zhang A. H. Zheng Q. L. Li P. C. Wang Y. Q. He L. M. (2024). The biomechanical effects of different membrane layer structures and material constitutive modeling on patient-specific cerebral aneurysms. Front. Bioeng. Biotechnol. 11, 1323266. 10.3389/fbioe.2023.1323266 38288243 PMC10822973

[B7] Jiang B. W. Paff M. Colby G. P. Coon A. L. Lin L. M. (2016). Cerebral aneurysm treatment: modern neurovascular techniques. Stroke Vasc. Neurol. 1 (3), 93–100. 10.1136/svn-2016-000027 28959469 PMC5435202

[B8] Jun Y. J. Hwang D. K. Lee H. S. Kim B. M. Park K. D. (2024). Flow diverter performance comparison of different wire materials for effective intracranial aneurysm treatment. Bioengineering 11 (1), 76. 10.3390/bioengineering11010076 38247953 PMC10813681

[B9] Kim J. H. Kang T. J. Yu W. R. (2008). Mechanical modeling of self-expandable stent fabricated using braiding technology. J. Biomech. 41 (15), 3202–3212. 10.1016/j.jbiomech.2008.08.005 18804764

[B10] Li Y. J. Zhang M. Z. Verrelli D. I. Chong W. Ohta M. Qian Y. (2018). Numerical simulation of aneurysmal haemodynamics with calibrated porous-medium models of flow-diverting stents. J. Biomech. 80, 88–94. 10.1016/j.jbiomech.2018.08.026 30190083

[B11] Ma J. Y. You Z. Byrne J. Rizkallah R. R. (2014). Design and mechanical properties of a novel cerebral flow diverter stent. Ann. Biomed. Eng. 42, 960–970. 10.1007/s10439-013-0967-3 24449051

[B12] Maragkos G. A. Dmytriw A. A. Salem M. M. Tutino V. M. Meng H. Cognard C. (2020). Overview of different flow diverters and flow dynamics. Neurosurgery 86, S21–S34. 10.1093/neuros/nyz323 31838536

[B13] Masuda S. Fujimura S. Takao H. Takeshita K. Suzuki T. Uchiyama Y. (2022). Effects of different stent wire mesh densities on hemodynamics in aneurysms of different sizes. PLoS One 17 (6), e0269675. 10.1371/journal.pone.0269675 35687558 PMC9187070

[B14] Murayama Y. Fujimura S. Suzuki T. Takao H. (2019). Computational fluid dynamics as a risk assessment tool for aneurysm rupture. Neurosurg. Focus 47 (1), E12. 10.3171/2019.4.focus19189 31261116

[B15] Mut F. Aubry R. Löhner R. Cebral J. R. (2010). Fast numerical solutions of patient‐specific blood flows in 3D arterial systems. Int. J. Numer. Methods Biomed. Eng. 26 (1), 73–85. 10.1002/cnm.1235 21076685 PMC2978074

[B16] Nada A. Hassan M. A. Fakhr M. A. El-Wakad M. T. I. (2021). Studying the effect of stent thickness and porosity on post-stent implantation hemodynamics. J. Med. Eng. Technol. 45 (5), 408–416. 10.1080/03091902.2021.1912204 33945392

[B17] Nada A. Fakhr M. A. El-Wakad M. T. I. Hassan M. A. (2022). A finite element-based analysis of a hemodynamics efficient flow stent suitable for different abdominal aneurysm shapes. J. Biomech. Eng. 144 (9), 091006. 10.1115/1.4053999 35237800

[B18] Nada A. Fakhr M. A. Ei-wakad M. T. I. Hassan M. A. (2025). Hemodynamic performance of three flow diverting stents for treatment of abdominal aortic aneurysm based on a simplified patient-specific model: a comparison study. J. Mech. Med. Biol. 25 (04), 2450051. 10.1142/s0219519424500519

[B19] Reorowicz P. Tyfa Z. Obidowski D. Wisniewski K. Stefanczyk L. Józwik K. (2022). Blood flow through the fusiform aneurysm treated with the flow diverter stent–numerical investigations. Biocybern. Biomed. Eng. 42 (1), 375–390. 10.1016/j.bbe.2022.02.008

[B20] Sindeev S. Arnold P. G. Frolov S. Prothmann S. Liepsch D. Balasso A. (2018). Phase-contrast MRI *versus* numerical simulation to quantify hemodynamical changes in cerebral aneurysms after flow diverter treatment. PLoS One 13 (1), e0190696. 10.1371/journal.pone.0190696 29304062 PMC5755883

[B21] Suzuki T. Takao H. Fujimura S. Dahmani C. Ishibashi T. Mamori H. (2017a). Relationships between geometrical parameters and mechanical properties for a helical braided flow diverter stent. Technol. Health Care 25 (4), 611–623. 10.3233/thc-160535 28506004

[B22] Suzuki T. Takao H. Fujimura S. Dahmani C. Ishibashi T. Mamori H. (2017b). Selection of helical braided flow diverter stents based on hemodynamic performance and mechanical properties. J. Neurointerv Surg. 9 (10), 999–1005. 10.1136/neurintsurg-2016-012561 27646987 PMC5629929

[B23] Tawk R. G. Hasan T. F. D`Souza C. E. Peel J. B. Freeman W. D. (2021). Diagnosis and treatment of unruptured intracranial aneurysms and aneurysmal subarachnoid hemorrhage. Mayo Clin. Proc. 96 (7), 1970–2000. 10.1016/j.mayocp.2021.01.005 33992453

[B24] Uchiyama Y. Fujimura S. Takao H. Suzuki T. Hayakawa M. Ishibashi T. (2021). Hemodynamic investigation of the effectiveness of a two overlapping flow diverter configuration for cerebral aneurysm treatment. Bioengineering 8 (10), 143. 10.3390/bioengineering8100143 34677216 PMC8533189

[B25] Wang C. Tian Z. B. Liu J. Jing L. K. Paliwal N. Wang S. Z. (2016). Flow diverter effect of LVIS stent on cerebral aneurysm hemodynamics: a comparison with enterprise stents and the pipeline device. J. Transl. Med. 14, 199–10. 10.1186/s12967-016-0959-9 27370946 PMC4930570

[B26] Wei L. L. Leo H. L. Chen Q. Li Z. Y. (2019). Structural and hemodynamic analyses of different stent structures in curved and stenotic coronary artery. Front. Bioeng. Biotechnol. 7, 366. 10.3389/fbioe.2019.00366 31867313 PMC6908811

[B27] Xiang Y. X. Zhang P. Lai Y. J. Wang D. H. Liu A. C. (2025). Risk factors, antithrombotic management, and long-term outcomes of patients undergoing endovascular treatment of unruptured intracranial aneurysms. Thromb. Haemost. 125 (01), 058–068. 10.1055/a-2347-4221 38889891

[B28] Zaccaria A. Migliavacca F. Pennati G. Petrini L. (2020). Modeling of braided stents: Comparison of geometry reconstruction and contact strategies. J. Biomech. 107, 109841. 10.1016/j.jbiomech.2020.109841 32517859

[B29] Zhang M. Z. Anzai H. Chopard B. Ohta M. (2016). Towards the patient-specific design of flow diverters made from helix-like wires: an optimization study. Biomed. Eng. Online 15, 159–382. 10.1186/s12938-016-0257-z 28155683 PMC5260143

[B30] Zhang Y. Wang Y. Kao E. Flórez-Valencia L. Courbebaisse G. (2019). Towards optimal flow diverter porosity for the treatment of intracranial aneurysm. J. Biomech. 82, 20–27. 10.1016/j.jbiomech.2018.10.002 30381156

[B31] Zhang K. Li T. X. Wang Z. L. Gao B. L. Gu J. J. Gao H. L. (2021). Factors affecting in-stent restenosis after angioplasty with the enterprise stent for intracranial atherosclerotic diseases. Sci. Rep. 11 (1), 10479. 10.1038/s41598-021-89670-x 34006896 PMC8131349

[B32] Zhao X. M. Li R. Chen Y. Sia S. F. Li D. H. Zhang Y. (2017). Hemodynamic analysis of intracranial aneurysms using phase-contrast magnetic resonance imaging and computational fluid dynamics. Acta Mech. Sin. 33 (2), 472–483. 10.1007/s10409-017-0636-0

[B33] Zhao Z. K. Qiu H. C. Song X. W. Xu X. B. Dong Y. Y. (2023). Effects of a novel stereoscopic flow-diverting stent on intracranial aneurysm hemodynamics. J. Biomed. Nanotechnol. 19 (3), 442–448. 10.1166/jbn.2023.3541

[B34] Zheng Q. L. Dong P. F. Li Z. Q. Han X. W. Zhou C. C. An M. W. (2019). Mechanical characterizations of braided composite stents made of helical polyethylene terephthalate strips and NiTi wires. Nanotechnol. Rev. 8 (1), 168–174. 10.1515/ntrev-2019-0016 35966892 PMC9368628

